# Ascorbic Acid Alleviates Water Stress in Young Peach Trees and Improves Their Performance after Rewatering

**DOI:** 10.3389/fpls.2017.01627

**Published:** 2017-09-20

**Authors:** Consuelo Penella, Ángeles Calatayud, Juan C. Melgar

**Affiliations:** ^1^Departamento de Horticultura, Instituto Valenciano de Investigaciones Agrarias Valencia, Spain; ^2^Department of Plant and Environmental Sciences, Clemson University, Clemson SC, United States

**Keywords:** biochemicals, water deficit, rewatering, gas exchange, water relations, lipid peroxidation

## Abstract

Exogenous application of biochemicals has been found to improve water stress tolerance in herbaceous crops but there are limited studies on deciduous fruit trees. The goal of this research was to study if ascorbic acid applications could improve physiological mechanisms associated with water stress tolerance in young fruit trees. Ascorbic acid was foliarly applied at a concentration of 250 ppm to water-stressed and well-watered peach trees (control) of two cultivars (‘Scarletprince’ and ‘CaroTiger’). Trees received either one or two applications, and 1 week after the second application all trees were rewatered to field capacity. Upon rewatering, CO_2_ assimilation and stomatal conductance of water-stressed ‘Scarletprince’ trees sprayed with ascorbic acid (one or two applications) were similar to those of well-irrigated trees, but water-stressed trees that had not received ascorbic acid did not recover photosynthetical functions. Also, water status in sprayed water-stressed ‘Scarletprince’ trees was improved to values similar to control trees. On the other hand, water-stressed ‘CaroTiger’ trees needed two applications of ascorbic acid to reach values of CO_2_ assimilation similar to control trees but these applications did not improve their water status. In general terms, different response mechanisms to cope with water stress in presence of ascorbic acid were found in each cultivar, with ‘Scarletprince’ trees preferentially using proline as compatible solute and ‘CaroTiger’ trees relying on stomatal regulation. The application of ascorbic acid reduced cell membrane damage and increased catalase activity in water-stressed trees of both cultivars. These results suggest that foliar applications of ascorbic acid could be used as a management practice for improving water stress tolerance of young trees under suboptimal water regimes.

## Introduction

Water availability for irrigation is among the most critical factors that affect fruit tree growth in commercial orchards and tree production in nurseries, especially considering the long-term and costly investments of planting fruit tree orchards. Water stress promotes stomatal closure and reduction in CO_2_ fixation that may cause over-reduction of the photosynthetic electron chain. If water stress is prolonged and/or severe, part of the energy supplied by incident photons may be redirected into processes that favor the generation of reactive oxygen species (ROS) such as hydrogen peroxide (H_2_O_2_), leading to oxidative damage to plant tissues (Jiang and Zhang, 2002). Nevertheless, plants can activate ROS-scavenging enzymes such as superoxide dismutase, catalase, ascorbate peroxidase or glutathione reductase, and non-enzymatic systems including secondary metabolites such as phenolic compounds, alkaloids, isoprenoids, phenylpropanoids, and other antioxidants such as glutathione and ascorbic acid (AsA) to reduce oxidative damage (Gill and Tuteja, 2010; Tattini et al., 2015). Furthermore, these systems can also play very important roles in the protection of cell membrane integrity (Khan et al., 2011; Patade et al., 2012).

Ascorbic acid and the ascorbate-glutathione pathway play an important role in detoxification of ROS and modulating other fundamental functions in plants under stress conditions (Gill and Tuteja, 2010; Foyer and Noctor, 2011; Akram et al., 2017). Ascorbic acid is also the major non-enzymatic antioxidant in the apoplast (Pignocchi and Foyer, 2003) where it also has a key role in environmental stress perception and stress signaling (Wolucka et al., 2005; Shapiguzov et al., 2012). Enhanced antioxidant systems induced by water stress have been described in herbaceous plants, for instance elevated AsA and increased activities of antioxidant enzymes in maize (Dolatabadian et al., 2009), or tomato (Murshed et al., 2013), and in fruit trees such as mandarin (Sarkar et al., 2016).

Plant tolerance to environmental stresses can be enhanced by the exogenous application of beneficial molecules such as proline, amino acids, humic acid, and other antioxidants (Hasanuzzaman et al., 2015). The physiological responses of herbaceous plants under water stress to exogenous AsA have been thoroughly studied (Athar et al., 2008; Dolatabadian et al., 2009; Malik and Ashraf, 2012; Xu et al., 2015; Mukhtar et al., 2016). Nevertheless, the effects of exogenous applications of AsA in fruit tree species under water stress have been scarcely researched [only in olive trees (Ibrahim, 2013) and grapevines (Zonouri et al., 2014)], and as of now, there are no studies on the effects of exogenous AsA on water-stressed deciduous fruit trees and their responses after rewatering.

Water stress can impair growth and entry into production of young fruit trees, and reduce growth, yield and fruit quality of mature trees. Water stress can be detrimental for young trees during the first year after planting in the field, especially in areas where the current practice is to start irrigation during the second year, as is commonly done in the Southeastern United States. In a previous study, young peach trees subjected to water stress for a prolonged period of time did not improve physiological indicators such as tree water status and sap flow rates even 1 week after irrigation is resumed (Remorini and Massai, 2003). The response to rewatering is especially relevant in young trees, for instance in nursery trees where water stress or even temporary irrigation problems could affect budding success and reduce maximum development rates and consequently nursery productivity.

Since exogenous AsA has been reported to positively impact physiological parameters in herbaceous plants under water stress, it was hypothesized that AsA applications may also improve responses of deciduous fruit trees to suboptimal water conditions. Thus, the goal of this research was to evaluate the efficacy of exogenous AsA applications in improving physiological responses (gas exchange and water relations, stomatal density and size, proline concentration, catalase activity and lipid peroxidation) of peach trees during water stress and upon rewatering.

## Materials and Methods

### Tree Growth Conditions

Fifty five 1-year-old peach trees [*Prunus persica* (L.) Batsch cvs. Scarletprince and CaroTiger] were grown into 11.4-L pots filled with a mixture of 7.6 L of potting soil (Fafard 3B Mix, Sun Gro Horticulture, Agawam, MA, United States) containing Canadian sphagnum peat moss, bark, perlite, vermiculite and dolomitic limestone, 3.8 L of sand, and 40 g of slow release fertilizer 14-14-14 (Osmocote^TM^, Scotts, Marysville, OH, United States). There were 26 ‘Scarletprince’ trees and 29 ‘CaroTiger’ trees, all grafted onto Guardian^TM^ rootstock. In October 2014, they were placed in a glass greenhouse with temperatures between 15.6°C (minimum) and 26.7°C (maximum), relative humidity between 34 and 57%, and natural photoperiods of ∼10.5 h. Trees were watered to field capacity every 3 days until the beginning of January 2015 (they did not shed their leaves during winter), when half of the trees of each cultivar stopped receiving water in order to establish two irrigation regimes: control trees (irrigated to field capacity every 3 days) and water-stressed trees (no application of water). Volumetric soil water content was measured daily with a 20-cm long time-domain reflectometry (TDR; FieldScout 100, Spectrum Tech., Aurora, IL, United States) probe.

Exogenous applications (foliar spray) of AsA at a concentration of 250 ppm were performed. Applications started when the average volumetric water content in the pots of water-stressed trees was 30%, which was more than 50% lower than the values observed in the well-watered trees (volumetric water content in control trees at field capacity averaged 63%). At this time, the following treatments were established: (1) Well-watered trees without AsA (untreated control); (2) well-watered trees with AsA (treated control); (3) water-stressed trees without AsA; (4) water-stressed trees that received one application of AsA; and (5) water-stress trees that received two applications of ascorbic acid (i.e., same as treatment 4 plus another application of AsA the following week). Ascorbic acid was applied at dusk to avoid light and warm temperatures. One week after the second application of AsA, all trees were rewatered to saturation.

### Gas Exchange and Water Relations

Gas exchange parameters were determined in the morning (9:00–11:00) to avoid midday depression of net gas exchange (Jifon and Syvertsen, 2003). Measurements were performed 16, 40, and 88 h and 1 week after the second application of AsA (this measurement was taken right before rewatering), and the day after rewatering (+24 h). Fully expanded leaves of five trees per treatment were used. Net CO_2_ assimilation rate (A_CO2_), stomatal conductance (g_s_), leaf transpiration (E_leaf_) and leaf water use efficiency (WUE_leaf_, calculated as A_CO2_ and E_leaf_) were measured with a photosynthesis system (LI-6400XTR; Licor, Lincoln, NE, United States). All these measurements were taken at a photosynthetically active radiation of 1,000 μmol m^-2^ s^-1^. Leaf temperature was 26 ± 6°C, and leaf-to-air water vapor pressure difference was 2.6 ± 1.6 kPa.

Midday stem water potential measurements (Ψ_w_) were taken in three leaves per treatment immediately before rewatering, 1 week after the second treatment with AsA. Before measuring, leaves had been introduced in a reflective plastic bags for over 1 h, and Ψ_w_ was measured with a pressure chamber (PMS 600, PMS Instruments, Albany, OR, United States; Scholander et al., 1965).

### Stomatal Density and Size

Three fully developed leaves per treatment and genotype were used for stomatal size and density determination, and two different areas per leaf were analyzed before rewatering, 1 week after the second AsA application. Transparent acrylic nail varnish was applied to the underside of leaves. A few minutes later, when the varnish was dried, transparent packing tape was used to peel off the nail polish. Each tape was placed onto a microscope slide, and observed at ×100 magnification (Olympus BX41, Olympus, Center Valley, PA, United States). Two areas of 10 mm^2^ from each slide were randomly selected and images were captured with a digital camera (Canon EOS Rebel T3i, Canon, Melville, NY, United States). Stomata were counted, and length and width of the ostioles were determined using ImageJ (1.48 v, National Institutes of Health, Bethesda, MD, United States).

### Proline, Catalase Activity, and Lipid Peroxidation Determinations

Proline concentration, catalase activity, and lipid peroxidation were measured in extracts obtained from five trees per treatment (one leaf per tree), 1 week after the second application of AsA. Samples for proline and catalase were collected immediately before rewatering, and samples for lipid peroxidation were collected after rewatering. Proline concentration was determined as in Bates et al. (1973), using 0.05 g fresh weight leaf tissue. Catalase activity was determined by the decomposition of H_2_O_2_ (Aebi, 1984). Lipid peroxidation was estimated by quantifying malondialdehyde (MDA) using the thiobarbituric acid reaction as in Penella et al. (2015).

### Data Analyses

Data were subjected to ANOVA (Statistix 8.0, Analytical Software, Tallahassee, FL, United States) as completely randomized design. When a significant *F*-test was observed, means were separated using Tukey’s test (*P* ≤ 0.05).

## Results

### Effects of Water Stress and AsA Sprayed on Gas Exchange Parameters and Water Relations

**FIGURE 1:**
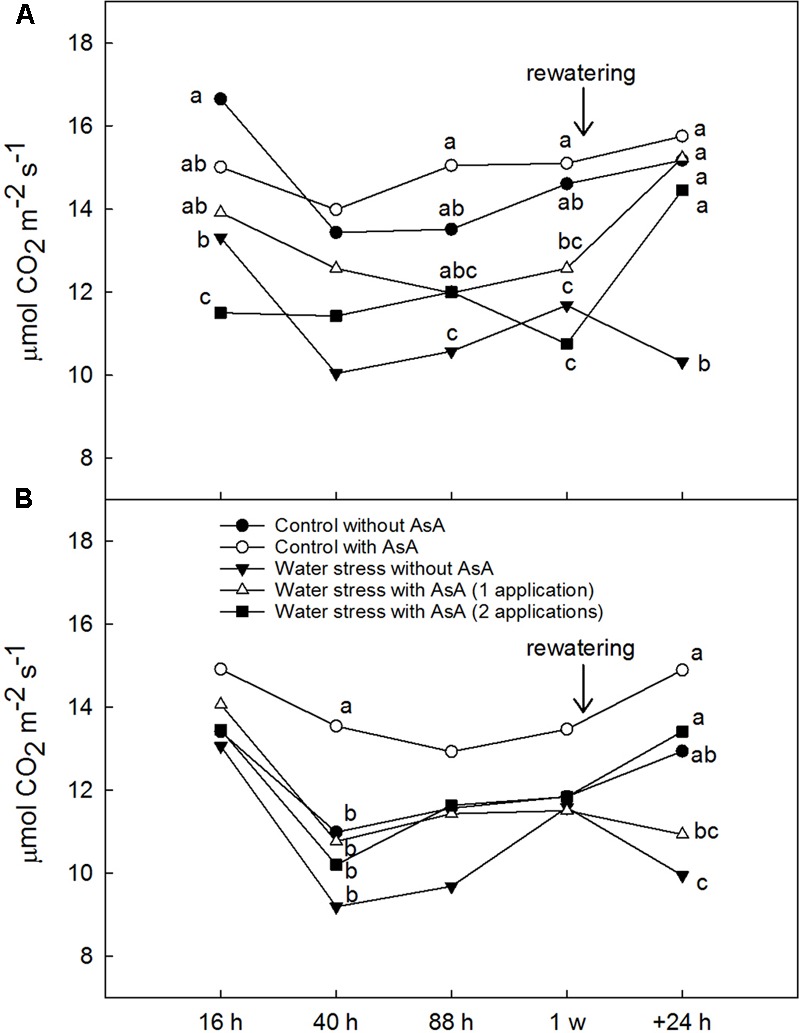
Net CO_2_ fixation rate (A_CO2_, μmol CO^2^ m^-2^ s^-1^) was measured on fully expanded leaves of ‘Scarletprince’ **(A)** and ‘CaroTiger’ **(B)** trees at steady-state conditions of saturating light (1,000 μmol m^-2^s^-1^) and 400 ppm CO_2_. Measurements were performed 16, 40, 88 h, 1 week after the second application of AsA (immediately before rewatering), and the day after rewatering (+24 h). Data are mean values for *n* = 5. For each time studied, different letters indicate significant differences at *P* ≤ 0.05 (Tukey’s test), following a one-way ANOVA test with treatment as variability factor.

**FIGURE 2:**
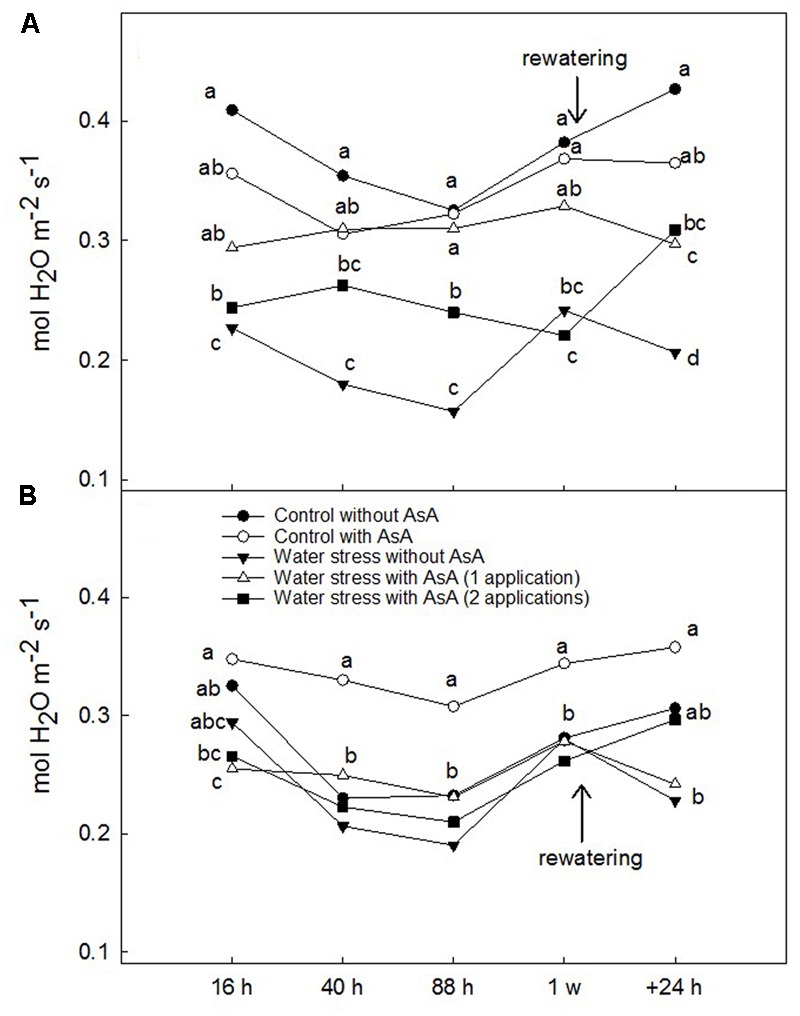
Stomatal conductance to water vapor (g_s_, mol H_2_O m^-2^ s^-1^) was measured on fully expanded leaves of ‘Scarletprince’ **(A)** and ‘CaroTiger’ **(B)** trees at steady-state conditions of saturating light (1,000 μmol m^-2^s^-1^) and 400 ppm CO_2_. Measurements were performed 16, 40, 88 h, 1 week after the second application of AsA (immediately before rewatering), and the day after rewatering (+24 h). Data are mean values for *n* = 5. For each time studied, different letters indicate significant differences at *P* ≤ 0.05 (Tukey’s test), following a one-way ANOVA test with treatment as variability factor.

Upon rewatering, water-stressed young ‘Scarletprince’ peach trees without AsA application showed lower A_CO2_ and g_s_ than well-watered (control) trees but trees that received one or two applications of AsA had A_CO2_ values similar to control trees and g_s_ values significantly higher than water-stressed trees without AsA (**Figures 1A, 2A**). On the other hand, water-stressed ‘CaroTiger’ trees that had received two AsA spray applications increased A_CO2_ and g_s_ upon rewatering to values comparable to those from control trees; however, one application of AsA was not effective to improve A_CO2_ or g_s_ (**Figures 1B, 2B**). Ascorbic acid did not have any effect on E_leaf_ or WUE_leaf_ in any of the cultivars (data not shown). However, WUE_leaf_ in ‘Scarletprince’ trees was significantly higher than in ‘CaroTiger’ trees, with mean WUE_leaf_ values of 3.2 and 2.5 μmol CO_2_ mol^-1^ H_2_O, respectively.

**FIGURE 3:**
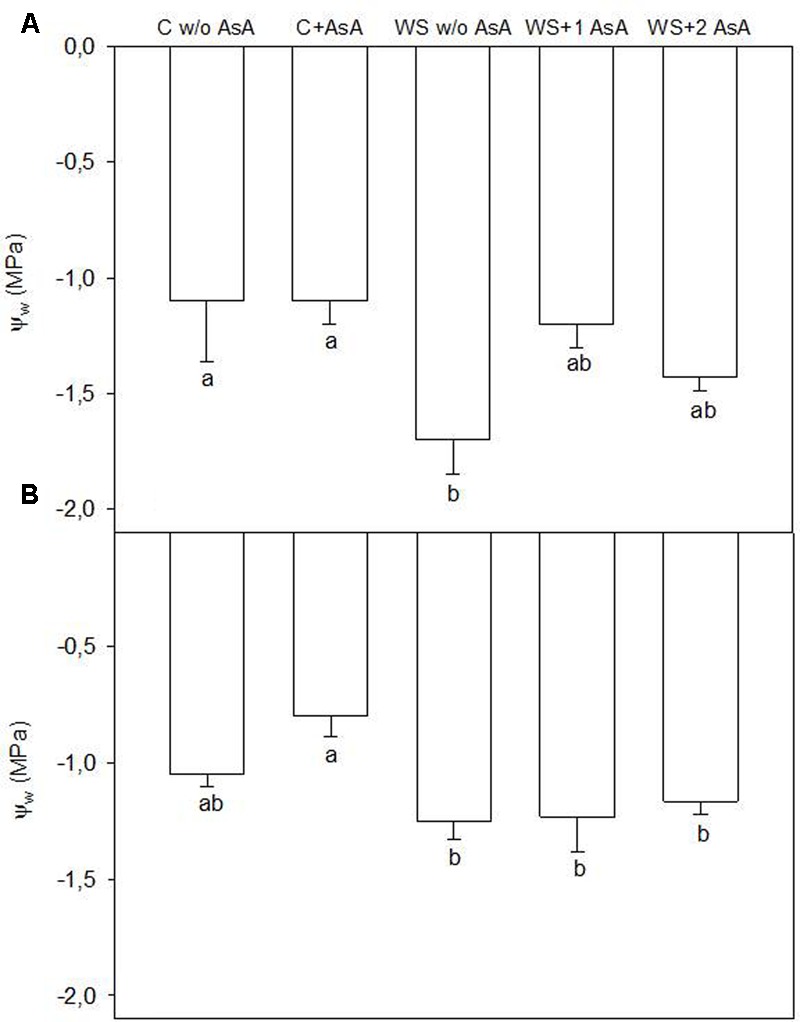
Water stem potential Ψ_w_ (MPa) was measured on fully expanded leaves of ‘Scarletprince’ **(A)** and ‘CaroTiger’ **(B)** trees. Measurements were performed 1 week after the second application of AsA (immediately before rewatering) onto plants grown under control conditions without and with AsA application (C w/o AsA and C+AsA, respectively), and under water stress conditions without AsA (WS w/o AsA), with only one AsA application (WS+1 AsA) and two AsA applications (WS+2 AsA). Data are mean values ± SE for *n* = 3. For each cultivar studied, different letters indicate significant differences at *P* ≤ 0.05 (Tukey’s test), following a one-way ANOVA test with treatment as variability factor.

Water-stressed ‘Scarletprince’ trees that had been sprayed with AsA had Ψ_w_ values similar to well-irrigated trees right before rewatering whereas water-stressed trees without AsA showed significantly lower values than irrigated trees (**Figure 3A**). However, all water-stressed ‘CaroTiger’ trees, independently of the AsA treatment, had similar Ψ_w_ and did not show differences compared to control trees without AsA application (**Figure 3B**).

### Stomatal Density and Size

Treatments did not affect stomatal density in any of the cultivars studied (data not shown). Ostiole length, width and area of water-stressed ‘Scarletprince’ trees that had not been sprayed with AsA were significantly smaller than those in leaves of control trees; however, stomata in leaves of water-stressed ‘Scarletprince’ trees sprayed with AsA had ostiole length, width and area similar to those in control trees, independently of numbers AsA applications (**Table 1**). Likewise, stomata in water-stressed ‘CaroTiger’ trees without AsA applications had smaller width and area than those of the other treatments, and stomata size in water-stressed trees with AsA application was similar to that in leaves from control trees. Interestingly, even though ostiole area values of water-stressed AsA-treated trees of both cultivars were similar to those of control trees, water-stressed ‘Scarletprince’ trees with AsA tended to have smaller ostiole area values than control trees whereas water-stressed ‘CaroTiger’ trees with AsA tended to have greater values than control trees. Then, when a statistical analysis comparing the effect of cultivar was run, ostiole area values of water-stressed ‘CaroTiger’ trees with AsA were greater than those of water-stressed ‘Scarletprince’ trees with AsA (data not shown).

**Table 1:** Effect of treatments on ostiole length (μm), width (μm), and area (μm^2^) from randomly selected areas (*n* = 2) in leaves (*n* = 3) of ‘Scarletprince’ and ‘CaroTiger’ trees.

	Scarletprince			CaroTiger		
	Length	Width	Area	Length	Width	Area
Control, no AsA	24.1 a^‡^	9.4 a	723 a	22.7	9.5 a	688 a
Control with AsA	22.1 ab	8.4 a	584 a	23.0	8.3 a	612 ab
Water-stressed, no AsA	20.0 b	5.6 b	347 b	22.1	5.6 b	406 b
Water-stressed with AsA (1)	22.4 ab	8.5 a	597 a	25.6	10.0 a	800 a
Water-stressed with AsA (2)	21.3 ab	9.0 a	607 a	23.6	9.6 a	737 a
Significance^†^	*^∗^*	^∗∗^	*^∗∗^*	N.S.	*^∗∗^*	^∗^

*^∗^*P* ≤ 0.05; ^∗∗^*P* ≤ 0.01; ^†^N.S., non-significant; ^‡^different letters indicate significant differences at *P* ≤ 0.05.*

### Effects on Proline

**FIGURE 4:**
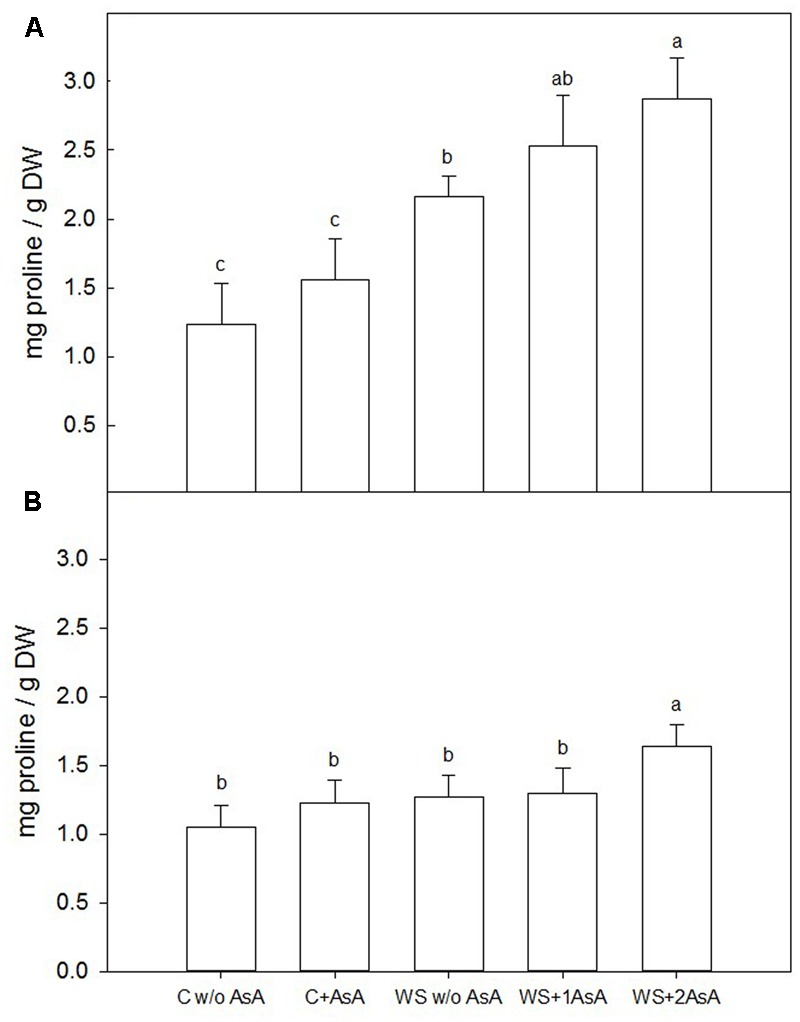
Changes in the proline concentration were measured on fully expanded leaves of ‘Scarletprince’ **(A)** and ‘CaroTiger’ **(B)** trees. Measurements were performed 1 week after the second application of AsA (immediately before rewatering) onto plants grown under control conditions without and with AsA application (C w/o AsA and C+AsA, respectively), and under water stress conditions without AsA (WS w/o AsA), with only one AsA application (WS+1 AsA) and two AsA applications (WS+2 AsA). Data are mean values ± SE for *n* = 5. For each cultivar studied, different letters indicate significant differences at *P* ≤ 0.05 (Tukey’s test), following a one-way ANOVA test with treatment as variability factor.

One week after the second AsA treatment (immediately before rewatering), water-stressed ‘Scarletprince’ trees showed higher proline concentration than well-watered trees, with trees treated twice with AsA showing the highest concentration, followed by those sprayed only one time and untreated water-stressed trees (**Figure 4A**). In ‘CaroTiger’ trees, exogenous AsA only increased proline concentration significantly in water-stressed trees with two AsA applications, whereas the other water-stressed trees showed similar proline concentrations to control trees (**Figure 4B**). However, the increased proline concentration in water-stressed ‘CaroTiger’ trees with two applications of AsA was remarkably small compared to the increase observed in ‘Scarletprince’ trees.

### Catalase and Lipid Peroxidation Effects under Water Stress and AsA Application

**FIGURE 5:**
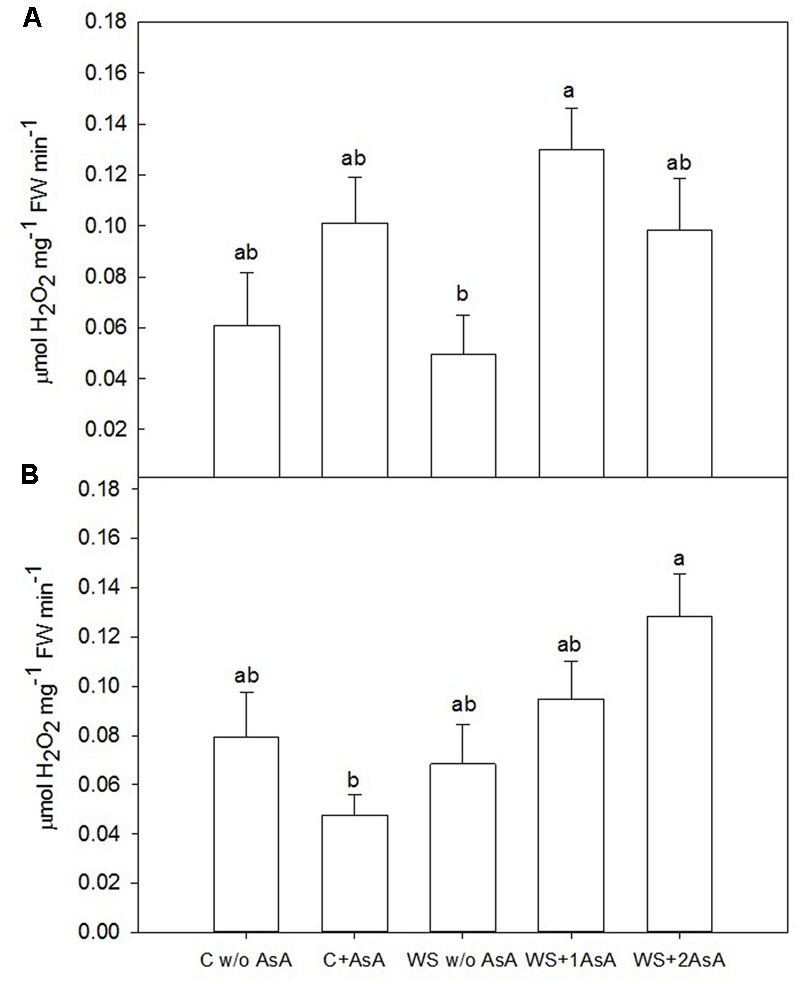
Catalase activity was measured on fully expanded leaves of ‘Scarletprince’ **(A)** and ‘CaroTiger’ **(B)** trees. Measurements were performed 1 week after the second application of AsA (immediately before rewatering) onto plants grown under control conditions without and with AsA application (C w/o AsA and C+AsA, respectively), and under water stress conditions without AsA (WS w/o AsA), with only one AsA application (WS+1 AsA) and two AsA applications (WS+2 AsA). Data are mean values ± SE for *n* = 5. For each cultivar studied, different letters indicate significant differences at *P* ≤ 0.05 (Tukey’s test), following a one-way ANOVA test with treatment as variability factor.

**FIGURE 6:**
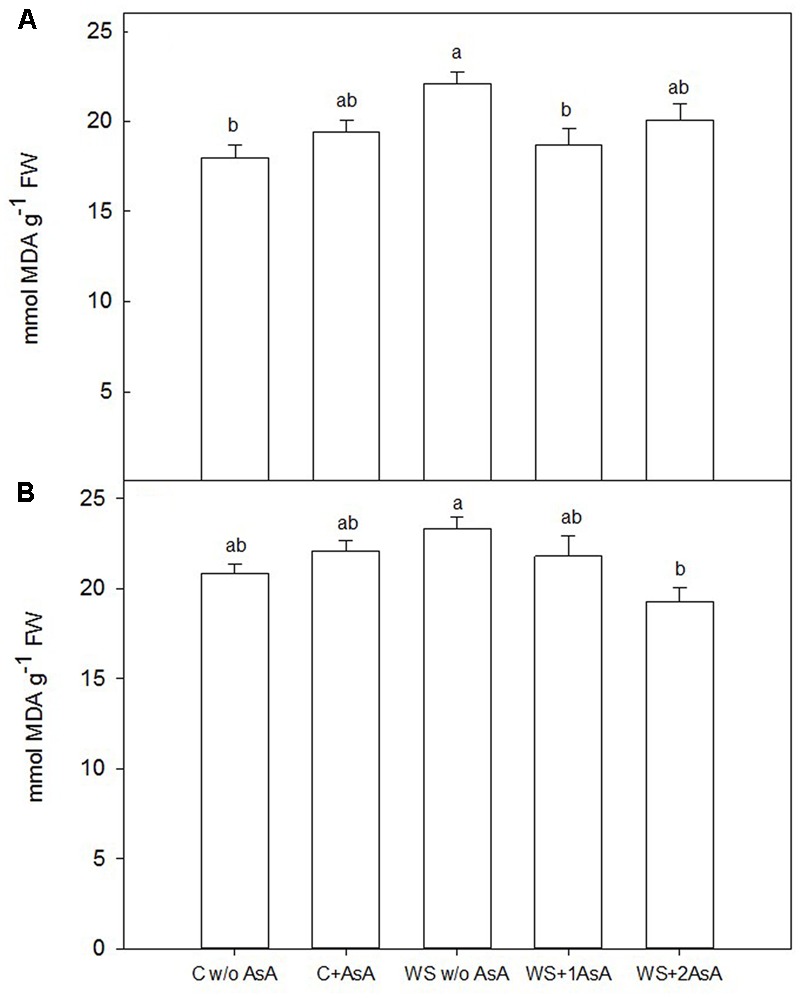
Malondialdehyde (MDA) was measured on fully expanded leaves of ‘Scarletprince’ **(A)** and ‘CaroTiger’ **(B)** trees. Measurements were performed 1 week after the second application of AsA (immediately before rewatering) onto plants grown under control conditions without and with AsA application (C w/o AsA and C+AsA, respectively), and under water stress conditions without AsA (WS w/o AsA), with only one AsA application (WS+1 AsA) and two AsA applications (WS+2 AsA). Data are mean values ± SE for *n* = 5. For each cultivar studied, different letters indicate significant differences at *P* ≤ 0.05 (Tukey’s test), following a one-way ANOVA test with treatment as variability factor.

In general terms, exogenous AsA increased catalase activity (**Figure 5**) and decreased peroxidation of lipids (**Figure 6**) in water-stressed trees of both cultivars. In water-stressed ‘Scarletprince’ trees, the treatment with one application of AsA was the most effective in increasing catalase activity (**Figure 5A**) and decreasing peroxidation of lipids (**Figure 6A**), whereas in water-stressed ‘CaroTiger’ trees, the treatment with two applications of AsA increased catalase activity (**Figure 5B**) and decreased peroxidation of lipids (**Figure 6B**) the most.

## Discussion

Ascorbic acid is the most abundant antioxidant in plants (Buettner and Jurkiewicz, 2006), and it has importance in photoprotection and regulation of photosynthesis though stomatal or non-stomatal factors (Foyer and Harbinson, 1994; Forti and Elli, 1995). In this study we demonstrated that foliar application of AsA in young peach trees could be a useful practice to overcome short periods of water shortage.

In regards to gas exchange, exogenous applications of AsA to young water-stressed peach trees significantly increased A_CO2_ upon rewatering in both cultivars to control levels. Biosynthesis of AsA occurs on the inner mitochondrial membrane through the oxidation of L-galactono-1,4-lactone (L-GalL). Recent research proved that exogenous applications of L-GalL to AsA-deficient Arabidopsis mutants increased A_CO2_, photosynthetic electron transport rate, and leaf stomatal conductance (Senn et al., 2016). Also, AsA has a role in photosynthesis and donates electrons to photosystems I and II when the primary electron donor system is damaged (Gallie, 2013). Net photosynthesis, growth rate and chlorophyll concentration also increased in wheat seedlings grown under water stress and supplemented with AsA in comparison with non-treated seedlings (Malik and Ashraf, 2012). The results observed in our experiment are in line with these studies since trees that received exogenous AsA had increased A_CO2_ values (after rewatering). This is of remarkable importance for alleviating the negative effects of water stress on photosynthesis reduction in young trees in commercial orchards undergoing a period of water stress (especially in areas where the current practice is to start irrigation after the second year on the ground), and in container-grown and field-grown young trees in tree nurseries, not only during periods of drought but also when field-grown nursery trees are dug and many fine roots are broken, which causes temporary water stress to trees until roots can reestablish.

In parallel with this increase in A_CO2_, exogenous AsA applications also increased g_s_ after rewatering. This view is supported by the roles of AsA in stomatal regulation (through the control of H_2_O_2_ levels that otherwise could trigger stomatal closure; Chen and Gallie, 2004) and in maintaining water homeostasis (Athar et al., 2008). These roles are especially important in trees under water stress. In our experiment, one application of AsA significantly increased g_s_ in water-stressed ‘Scarletprince’ trees during the experiment, and two applications increased g_s_ in water-stressed trees of both cultivars upon rewatering. Interestingly, water-stressed ‘Scarletprince’ trees with a single application of AsA showed a less negative Ψ_w_ before rewatering than water-stressed trees that had not received AsA, and water-stressed ‘Scarletprince’ trees with either one or two AsA applications showed increased proline concentration before rewatering (twofold values compared to control trees), which correspond with Ψ_w_ values in AsA-treated water-stressed trees similar to control trees. However, proline concentration did not increase in water-stressed ‘CaroTiger’ trees, and only water-stressed trees with two applications of AsA had a 0.5-fold increase in proline concentration, which did not improve tree Ψ_w_. The buildup of compatible solutes such as proline in water-stressed plants may contribute to the lowering of osmotic potential after rewatering and allow water to move into the cells (Tyree and Jarvis, 1982), which would partly explain the different responses of the two cultivars on gas exchange upon rewatering.

Furthermore, differences in ostiole area and WUE_leaf_ between cultivars were also found. Even though ostiole area of water-stressed trees with AsA applications was similar to that of control trees in both cultivars, water-stressed ‘Scarletprince’ trees with AsA applications had smaller ostiole area values than control (well-watered, no AsA) trees, whereas ‘CaroTiger’ water-stressed trees had greater area than control trees. This was probably the reason why ‘Scarletprince’ trees showed higher WUE_leaf_ than ‘CaroTiger’ trees (28% higher). Thus, both cultivars seem to have different response mechanisms when subjected to water stress, and these mechanisms seem to be controlled at the leaf level since both cultivars were grafted onto the same rootstock: accumulation of proline in ‘Scarletprince’ trees and stomatal control in ‘CaroTiger’. Different responses to AsA applications in cultivars of barley (Salama et al., 1994) and wheat (Athar et al., 2008) have also been reported.

The role of exogenous AsA on tree water status must be assessed because its ability to retain water under water stress conditions could improve tissue tolerance by protecting carboxylation, and inactivation and denaturation of enzymes (Anjum et al., 2012). For instance, our results show that even though each cultivar had different protective mechanisms, A_CO2_ values in water-stressed trees with two AsA applications was similar in both cultivars.

Photosynthesis reduction caused by water stress-induced reduction in g_s_ in trees without AsA exposes chloroplasts to excessive excitation energy and induces oxidative stress by increasing the production of ROS such as H_2_O_2_. H_2_O_2_ is an important signaling molecule that promotes closing of stomata (Pei et al., 2000; Murata et al., 2001). Plants with a high level of ascorbate are less responsive to H_2_O_2_ signaling (Chen and Gallie, 2004) and, because AsA scavenges H_2_O_2_, exogenous applications of AsA have been found to reverse H_2_O_2_-induced stomatal closing (Zhang et al., 2001). Studies performed in several *Prunus* hybrids showed that water stress caused H_2_O_2_-induced oxidative stress and increased activity of the ascorbate-glutathione cycle-associated enzymes and their antioxidant substrates; nevertheless, this was reversed upon rewatering (Sofo et al., 2005), which is in agreement with our results.

In addition, lipid peroxidation causes cell membrane damage including decreased fluidity and increased permeability. Malondialdehyde is a product of lipid peroxidation and, thus, it is commonly used to determine cell membrane damage caused by oxidative processes (Calatayud et al., 2002; Ozkur et al., 2009). Our results show that AsA applications could have modified the antioxidant capacity of the trees since AsA-treated trees showed lower MDA levels: AsA “*per se*” scavenges ROS and prevents protein oxidation and degradation (Noctor and Foyer, 1998) and, additionally, enzymatic antioxidant systems in AsA-treated trees were stimulated. In this sense, proline has been described to act as an inhibitor of lipid peroxidation in drought-stressed rice (Guo et al., 2005) and wheat (Farooq et al., 2013), and as a scavenger of ROS under different abiotic stresses (Gill and Tuteja, 2010). Thus, proline accumulation in water-stressed trees treated with AsA in our study may have also participated in reducing lipid peroxidation as shown by the low levels of MDA in sprayed trees.

A simultaneous activity of antioxidants is assumed to be necessary for a fast response when plants face water stress (Kwak et al., 2009). In our experiment, the activity of catalase, which is involved in removing and/or scavenging ROS, was significantly stimulated in AsA-treated trees of both cultivars. A similar increase in catalase activity was reported in wheat treated with AsA and grown under salt stress (Athar et al., 2008). Specifically in our experiment, one application of AsA only increased catalase activity in ‘Scarletprince’ trees whereas ‘CaroTiger’ trees needed two applications to reach a similar value. Although other enzymatic antioxidant systems that have not been measured in our experiment such as peroxidases may have accounted for elimination of hydrogen peroxide, non-sprayed water-stressed trees of both cultivars had increased MDA and decreased photosynthesis even after re-watering, which indicates that antioxidants did not efficiently maintain ROS scavenging in water-stressed trees that had not received AsA. This is supported by the fact that major detoxification of ROS produced during photosynthesis are mediated by catalase and by reductive processes involving major redox buffers such as ascorbate (Noctor and Foyer, 1998; Foyer and Noctor, 2003). Thus, our results imply an accumulation of ROS as a consequence of water stress and a reduction due to the increased catalase activity caused by the application of AsA. By contrast, the untreated stressed trees showed the lowest stem water potential values, which was probably linked to a minor accumulation of proline, less catalase activity, and increased lipid peroxidation.

## Conclusion

We can conclude that exogenous application of AsA could improve tree physiological responses to suboptimal water conditions and upon rewatering. Nevertheless, the parameters measured on water-stressed ‘Scarletprince’ and ‘CaroTiger’ trees revealed that each cultivar had a different response mechanism (the former using solute accumulation and the latter relying mainly on stomatal control), which can have implications on the potential use of exogenous biochemicals to improve water stress tolerance. Further applied research studies should explore the effect of applications of AsA at different concentrations on stress tolerance and recovery.

## Author Contributions

CP carried out the experiment under the supervision of ÁC (dissertation adviser) and JM (supervisor at Clemson University). Measurements were carried out by CP and JM. The paper was written and edited by the three authors.

## Conflict of Interest Statement

The authors declare that the research was conducted in the absence of any commercial or financial relationships that could be construed as a potential conflict of interest.
